# Developing and testing a community based, online vs. face-to-face peer led intervention to improve mental well-being in Cambodian adults with physical disabilities

**DOI:** 10.3389/fdgth.2024.1372062

**Published:** 2024-08-27

**Authors:** Paul Best, Alan Maddock, Nil Ean, Lorna Montgomery, Cherie Armour, Ciaran Mulholland, Carolyn Blair

**Affiliations:** ^1^ Centre for Technological Innovation, Mental Health and Education (TIME), Queen’s University Belfast, Belfast, United Kingdom; ^2^ Department of Health Psychology, School of Population Health, Royal College of Surgeons in Ireland, Dublin, Ireland; ^3^ Psychology Department, Royal University of Phnom Penh, Phnom Penh, Cambodia

**Keywords:** peer support, Cambodia, mental health, PTSD, friendship group

## Abstract

**Background:**

Despite growing international attention, there remains an urgent need to develop mental health services within low and middle income countries. The Khmer Rouge period in Cambodia saw the destruction of all health services infrastructure in the 1970s. Consequently, Cambodia has struggled to rebuild both its economy and healthcare system, with the number of qualified mental health clinicians remaining disproportionately low. Resultantly, there is a pressing need to develop low-cost community based alternatives of mental healthcare.

**Methods:**

Using a mixed methods design, researchers developed an 8-week peer-led intervention, known as a Friendship Group, for adults with physical disabilities using both face-to-face and online delivery methods. The Wilcoxon Signed-Rank test was used to assess changes in pre-post survey scores and qualitative data was collected in form of five focus groups post intervention.

**Results:**

41 participants were allocated across four Friendship groups – two were online and two face-to-face. Attrition rate was 22% post-intervention (*n* = 32). ITT analyses showed a statistically significant decrease in psychological distress scores [*Z* = −3.808, *p* < .001] from pre [Mdn = 20, IQR = 16.5–25.5] to post [Mdn = 16, IQR = 14–18.5] intervention. A Wilcoxon signed-ranks test also showed a statistically significant decrease in PTSD scores [*Z* = −2.239, *p* < .025] from pre [Mdn = 4, IQR = 3–5] to post [Mdn = 3, IQR = 2.75–4] intervention. There was also a statistically significant decrease in worry scores [*Z* = −3.904, *p* < .001] from pre [Mdn = 5, IQR = 3.5–6.5] to post [Mdn = 3, IQR = 3–4] intervention. There were no significant group differences between the face to face and online groups. A number of interconnected themes emerged from focus group data (*n* = 5), these included the mental health benefits of Friendship Groups as conceptualised through knowledge acquisition, skill development and peer support.

**Conclusions:**

The Friendship group intervention delivered in both online and face-to-face formats appears feasible and acceptable within the Cambodian context. Initial data revealed positive findings in terms of reduction in psychological distress, worry and PTSD symptoms as well increased feeling as calm.

## Introduction

Rates of mental health disorders in Cambodia are notably higher than in other low-or middle-income countries (1). Prevalence studies have estimated the probable rates of common mental health disorders in Cambodia stand at 16.7% for depression, 27.4% for anxiety and 7.6% for post-traumatic stress (PTSD) (2). Compared with global figures, this is approximately five times higher than the rest of the world (3). Following the Khmer Rouge period (1975–1979), when over one million people were killed, mental health services in Cambodia started to gradually re-emerge during the 1990s to tackle the crisis. Faced not only with a population living in the aftermath of genocide and trauma; mental health services were also confronted with a society struggling with inadequate access to food, financial stress, familial issues, and the fear of landmine injuries (4) However, the lack of resources and suitable infrastructure meant these mental health needs have largely gone unaddressed and unmet e.g., the number of psychiatrists and psychiatric nurses is approximately, 0.33 and 0.26 per 100,000 people (4). In addition, the total number of inpatient beds is 15, which is approximately 0.1 beds per 100,000 people (5). This is further compounded when one looks at the additional barriers to accessing community mental services for those with disabilities (6, 7). This is despite evidence showing elevated psychological distress and depression among Cambodia adults with physical disabilities (8).

Interventions for mental health disorders are clearly needed in Cambodia, however evidence suggests there is an absence of appropriate numbers of mental health professionals and mental health funding allocations (9). In recent years more Cambodian nationals are being trained as psychologists or mental health therapists, however the results of a qualitative study (*n* = 95) indicates that for these nationals, using Western-based therapy presents a myriad of challenges at individual, agency, family, cultural and community levels (10). It can be seen as infantilising, demeaning and ethnocentric when explaining westernised concepts of mental health. As such, there is a need for imported therapeutic models to be more culturally responsive to generate acceptance and impact in Cambodia (10). Innovative solutions are clearly required to address mental health issues in Cambodia. In particular, there is a definitive need for the Cambodian government to develop effective research and development strategies, and to devise a clear policy framework for health research to support projects which aim to train Cambodia nationals to deliver co-produced interventions to aid in psychosocial support (11–14). Given that international services addressing mental health issues have been criticized for cultural insensitivity and an inability to integrate with local communities (15), the best solutions are arguably those which are developed within the contexts in which they will operate (9). However, it remains unclear which mental health psychological and social interventions are the most effective in a Cambodian context augmented by the lack of cultural congruency and poor quality research studies (16). In a systematic review of psychological and social interventions for mental health issues and disorders in Southeast Asia, Maddock et al. (17), found some promising preliminary evidence that meditation and/or yoga could reduce the prevalence of symptoms of depression, anxiety and depressive disorders, and improve well-being in depressed clients (18, 19). These findings correlate with Cramer et al. (20) who conducted a systematic review of 12 Randomised Controlled Trials (RCTs) examining the effectiveness of mind–body yoga and relaxation interventions, however as Maddock et al. (17) state although the findings are encouraging more research and in particular RCTs of a higher quality are needed in a South East Asian context to consolidate the evidence base. Interestingly, Maddock et al. (17) also found that the highest quality, and most promising evidence came from the evaluations of lay delivered interventions. This evidence demonstrates the feasibility and potential sustainability of implementing such interventions in resource constrained contexts such as Cambodia (17). In light of the significant challenges facing mental health services in Cambodia, there is a need to develop low cost mental health interventions that could be delivered at scale within local communities by non-professionals (21). This process is known as “task shifting” and is recognised as a viable strategy for increasing access to mental health services for underserved populations (22–24).

Peer support in a mental health context can be described as involving people with personal experience of mental health problems in the delivery of support services (25). Peer support can include one to one support delivered by a person with experience of mental health problems, mutual support groups and peer-led mental health services (26). The uniqueness of peer support is that the individuals share similar characteristics or experiences as their peers within the group and thereby can create a connection which facilitates sharing of challenges and experiences as an “insider” (25). Several RCTs support the view that peer support programs can lead to improvements in empowerment, quality of life and depression and have a positive impact on hope and personal recovery (27, 28).

Ibrahim et al. (29) highlight that peer support significantly contributes to promoting recovery and reducing stigma through shared lived experiences, for example, Lyons et al. (30) conducted a systematic review and meta-analysis, including data from 2,131 participants across eight trials, showing that group peer support interventions lead to small but significant improvements in personal recovery outcomes, such as hope and empowerment. Further, Cooper et al. (31) conducted an umbrella review summarizing evidence from 35 reviews on peer support approaches. Despite mixed effectiveness results, their findings indicated that peer support might improve depression symptoms, self-efficacy, and recovery. They also identified factors promoting successful implementation, such as adequate training, supervision, a recovery-oriented workplace, and effective collaboration. As such, given the paucity of high-quality research and a heterogeneity between studies and types of peer support delivered it is challenging to conclude on the effectiveness of peer support (26, 32–34). It is also unclear as to whether these interventions will address depression or anxiety symptoms because of trauma. Nevertheless, given the findings of a recent systematic review and realist synthesis, peer support may be a viable option for low-middle income countries such as Cambodia as it is an economically viable way to engage underserved populations at a community level given the impact of historical traumas on community mental health (35).

This paper reports on the results of a mental health intervention development study using a mixed methods for Cambodian adults with physical disabilities using both face-to-face and online delivery methods. The study was designed by a multi-disciplinary and multinational collaboration between physical and mental health academics/practitioners across social work, psychology, physical rehabilitation (prosthetics and orthotics) and staff at the Cambodian School of Prosthetics and Orthotics (Exceed Worldwide). Exceed Worldwide trains Prosthetist Orthotists and Technicians at three separate clinics across Cambodia and provides free physical rehabilitation services, to those most in need.

### Aims and objectives

The aim of the study was to develop and implement a peer-led, group based, mental health intervention (known as Friendship Groups) for Cambodian adults with physical disabilities using both face-to-face and online delivery methods.

### Objectives

• RO1: To compare uptake and retention between face-to-face and online delivery, aiming for at least 70% enrollment of eligible participants and a dropout rate not exceeding 30%.
• RO2: To explore the potential benefit of weekly friendship groups in improving psychological distress.
• RO3: To collect preliminary efficacy data on the intervention's impact on psychological distress, PTSD symptoms, pathological worry, rumination, and facets of mindfulness, in preparation for a larger pilot RCT including a control group.
• RO4: To compare pre to post outcome data from those who attended the face-to-face group and those who attended the online group.
• RO5: To train (task-shift) local Cambodia staff that work in the Cambodian School of Prosthetics and Orthotics to deliver a weekly “friendship group” intervention for stress, low mood, and anxiety.
• RO6: To gather qualitative feedback from participants and facilitators to understand the acceptability and potential barriers to the intervention.
• RO7: To develop a logic model to inform intervention development and the evaluation of the intervention at a larger scale trial.

RO = Research Objective.

## Materials and methods

Using a mixed methods design, the research team developed and implemented an 8-week peer-led intervention (known as a Friendship Group (FG) for Cambodian adults with physical disabilities using both face-to-face and online delivery methods (Trial no: Trial Registration: NCT05725707 - retrospectively registered, 23/02/2023). Included within study objectives are a number of questions to explore whether a larger scale trial is feasible. As such, the design of this study is straddled between early intervention development and feasibility trial. Ethical approval was granted by the University's ethics committee (REF: 028_2021). Where relevant, the authors followed the CONSORT checklist for feasibility trials (36) as well as the guidance on complex intervention development by O'Cathain and colleagues (37) (see Table 1). Scores across both delivery methods were compared at baseline and post-intervention and included measures of psychological distress and PTSD as *outcomes*, and facts of mindfulness, worry and rumination as proposed *mediators* of change.

**Table 1:** Intervention development steps.

Action	Key task undertaken
Plan the development process	- Workshop with key stakeholders took place in Phnom Penh in Jan 2019 - Established mental health issues and lack of services as priority (literature and stakeholder feedback) - Built upon pre-existing knowledge of peer-led interventions (both within literature as well as practical experience within the team) - Funding secured from Global Challenges Research Fund - Protocol developed and ethical approval granted
Involve stakeholders	- Key stakeholders included as members of research team - Planning meetings held and work divided equally among partners (emphasis on using local researchers for data collection) - Links established/built with local University staff - Identification of local community workers to be trained as group leaders
Bring together a team and establish decision making processes	- International research team including those with expertise in intervention development, physical disability, mental health, CBT, mindfulness, social work, psychology and peer-led support groups - Shared decision making processes with academic partners leading research design components and clinical colleagues designing intervention content.
Review published research evidence	- Systematic review of psychological and social interventions for mental health issues completed in 2020 (published in 2021) - Qualitative study of lived experiences of target population also undertaken (published 2021)
Draw on existing theories	- Core theories identified in relation behavioural activation and mindfulness as potentially relevant within Cambodian context - Theories regarding peer-led support also considered given importance of informal or familial support networks within Cambodia culture
Articulate programme theory	- See logic model
Undertake primary data collection	- Both quantitative and qualitative data collections methods employed. - Quantitative data used to measure change in outcomes - Qualitative data used to understand context and explore issues in more depth
Understand context	- Consideration throughout of Cambodian context and different cultural attitudes and conceptualisations of mental health - Worked closely with academics, clinical staff and service providers from Cambodia to deliver the intervention - All intervention materials made available in Khmer and English
Pay attention to future implementation of the intervention to the real world	- Economic factors were considered from the outset. Local community workers trained to deliver intervention - All implementation of the peer-led intervention was carried out locally - Contingency plans in place for Covid-19 and post Covid-19 implementation
Design and refine the intervention	- Feedback gathered from group participants and group leaders/facilitators - Refinements made to protocol for implementing online groups and training for group leaders - Data gathered on acceptability - Initial data suggests mechanisms of action are supported
End the development phase	- Plans to transition onto controlled experimental design - Continued refinements to study protocol - Exploration of additional training content for group leader training

### Friendship group intervention (FG)

#### Development

The development of FG's were first discussed during thematically focused workshop in Cambodia in 2019. The aim of the workshops was to understand mental health issues in Cambodia, cultural differences and variance and different ways that mental health is conceptualised and responded to. During this workshop, members of the UK research team shared information regarding a local project upon which a peer-led support group model was being implemented. Consequently, a partnership of workshop attendees was consolidated with the aim of understanding how a peer support model could be adapted within a Cambodian context. This included Cambodian academics, mental health clinicians and a NGO as well as academics from the UK, Ireland and Sweden. The term “Friendship Group” was created to mitigate the potential impact of societal stigma on taking part in the intervention.

**Figure 1:**
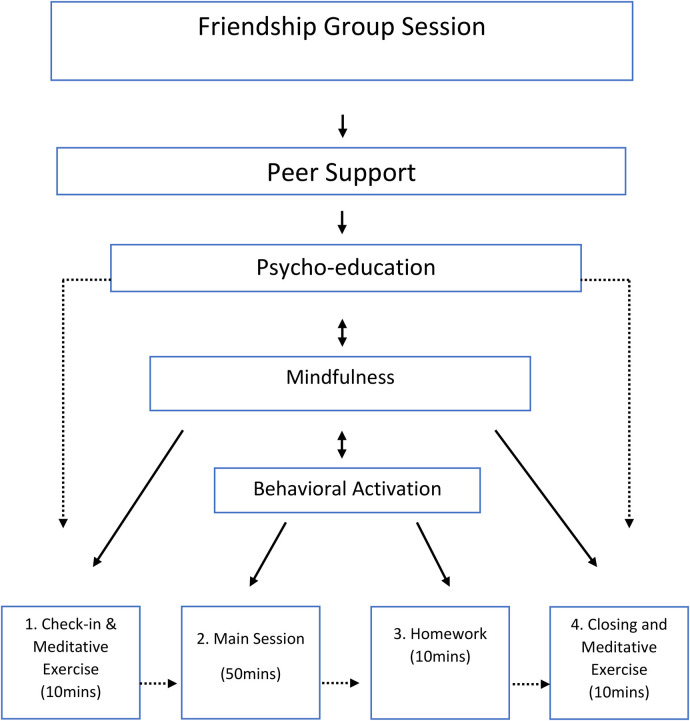
Friendship group components.

A template for peer support groups was provided by a community-based service in the UK and adapted for the Cambodian context. This support group template had been successfully delivered in both online and face-to-face formats in a previous research project (38). This included guidance on potential timing of groups (80 min) as well as advice on facilitation techniques, contracting, boundaries and safeguarding. Added to this were additional components, such as behavioural activation (39) and mindfulness-based practices (40–42). These components were selected due to their proven efficacy within other settings as well as their cultural relevance and likely acceptance within a Cambodian context. For example, mindfulness techniques are strongly aligned to the Buddhist informed approach to life which is prevalent in Cambodia (43–45). In addition, behavioural-based approaches, such as behavioural activation were believed to be more closely aligned to Cambodia perspectives on mental health issues. These are often described using physical, rather than cognitive or emotional terminology e.g., tiredness, restless, low energy (see Figure 1). Bernal et al. (46), provides a useful framework to inform the development of interventions, in terms of cultural relevance and acceptability. This includes eight key elements, such as language, persons, metaphors, content, concepts, goals, methods, and context. Adaptations outlined above were designed with these areas in mind. This included ensuring materials were translated appropriately into Khmer, that tasks/activities aligned with local cultural practices, and that they incorporated relevant metaphors and examples. A final important aspect was the inclusion of both UK and Cambodian academics as well as local service providers as members of the research team.

The development of the FG intervention also drew upon the mental health experience of the research team. This included those with clinical experience within the Cambodian mental health system as well as those from a range of professional backgrounds (social work, clinical psychologist, Cognitive Behavioural Therapist, and peer support group leader). The hypothesised change mechanisms within the FG's included: Psychoeducation; Peer support; Mindfulness and Behavioural activation. Whether face-to-face or online delivery, each FG session followed the same broad format - (1) Group meditative exercise and reminder of group agreement (10 min); (2) Check in with support group members and main session (50 min); (3) Summary of group discussion and homework activity planning (10 min); (4) Final group meditative exercise and closing of group (10 min). Session length was up to 80 min (see Table 2) and each facilitator was provided with a manual describing each section as well as some hints and tips to promote discussion and engagement. While all FG's would follow this structure, group members would drive the content (particularly during Step 2).

**Table 2:** Overview of stages within friendship groups.

	Stage	Time	Details
1	Group meditative exercise and reminder of group agreement	10 min	Task 1: members are asked to close their eyes if they feel comfortable doing so. If they do not, participants can gaze straight ahead, focusing on one point in front of them Task 2: they are encouraged to focus on their breathing, as air enters and leaves the nostrils – for 3 min approximately After 1 min, members will be asked to focus their attention on the crown of their head, and slowly the guide will gently encourage the participants to slowly move their attention from the crown of their head to their toes through: (a) their face – forehead, cheeks moving down to chin, (b) the neck, then the chest moving down to their stomach (noticing the air in the stomach as it rises up and descends), (c) then the hips, thighs and hamstrings, (d) moving down through the knees, shins, ankles and then toes and souls of their feet.
2	Check in with support group members and main session	50 min	The group leader will start with members on their left and move around in a circular manner – asking each person to firstly introduce themselves (their name and where they are from). After everyone has spoken a general question is asked to the whole group – “who would like to start to tell us how they have been doing today” Remembering what group members have said will allow you to ask follow up questions and look for common themes that may generate discussion. For example, “*I noticed that you had also mentioned problems in work in this week, on hearing Sok*'*s story do you think you could have approached things differently”* OR “*has anyone in the group felt the same way over the past week”.*
3	Summary of group discussion and homework activity planning	10 min	Task 1 – Agree a homework activity for everyone to do. Tips – • Be specific in terms of detail – time, place, activity and what is the potential benefit • Check that everyone understands what is being asked of them • Ask if there are any barriers that may prevent it from being completed Task 2 – Summary group discussion and thank members for their contribution.
4	Final group meditative exercise and closing of group	10 min	Task 1 - As the group begins to draw to a close it's important to summarise key discussion points and thank all members for attending. Even those who have not spoken have still contributed by listening and showing respect to the problems of their fellow members. Task 2 - Repeat meditative exercise to finish out the group

#### Delivery

Weekly Friendship Groups (*n* = 4) were delivered over an 8-week period (December 2021–January 2022) to individuals who met the clinical threshold as per Kessler-10 score (47). FG's were offered face-to-face in Phnom Penh (*n* = 2) and online via Zoom (*n* = 2). Trained, community support workers and Prosthetists’ from the Cambodian School of Prosthetics and Orthotics delivered FG's each week and followed the same four part structure described below. Prior to the first online session, each individual was asked to take part in a one-to-one orientation session to the platform to ensure that they could access the system without any issues. Participants were also reimbursed with costs for attending FG's (e.g., taxi fares for face-to-face or internet data for online groups). All those who had consented to receive communications were sent a weekly text message reminder 24 h prior to the next FG meeting. All sessions were delivered in the local language (Khmer).

### Recruitment

#### Recruitment and training of friendship group facilitators

FG facilitators were recruited from within the Cambodian School of Prosthetics and Orthotics (Exceed Worldwide) via internal expression of interest. Candidates with lived experience were encouraged to apply. Once identified (*n* = 10), facilitators took part in five days of training approximately one month before the intervention began. The role of the facilitator was to support and encourage group discussion as well as ensure that all FG's followed the same broad structure. Training was delivered by an experienced clinical psychologist who also provided weekly supervision during the 8-week intervention (see Table 3). Training included the following core topics –

**Table 3:** Friendship group facilitator training structure.

Friendship group facilitator training		
	Morning session	Afternoon session
Day one	Introduction to common mental health conditions in Cambodia	What is a Friendship Group?
Day two	Interpersonal skills development Working in groups	Role plays and feedback
Day three	Structure of Friendship Groups Delivering sessions online	Role plays and feedback
Day four	Dealing with disclosure and risk Self-care, supervision and boundaries	No session
Day five	Potential scenarios Group based facilitation practice	Role plays and feedback Final assessment

#### Recruitment of friendship group participants

As mentioned above, participants were drawn from those who were screened following a routine orthotic or prosthetic appointment. Screening items included the Kessler-10 psychological distress scale, which has been previously validated within the Cambodia context (48), and if attendees scored in the mild to moderate range on this scale (20–29), they were invited to register their interest in joining a Friendship group (47). Further information on inclusion criteria is given in Table 4.

**Table 4:** Inclusion and exclusion criteria.

Inclusion criteria	Exclusion criteria
Patients and prospective patients attending Cambodian School of Prosthetics and Orthotics	Actively suicidal
Meet the clinical threshold during Stage 1 screening	In receipt of additional specialist psychological therapy
Adults over the age of 18	Unable to give informed consent

**Figure 2:**
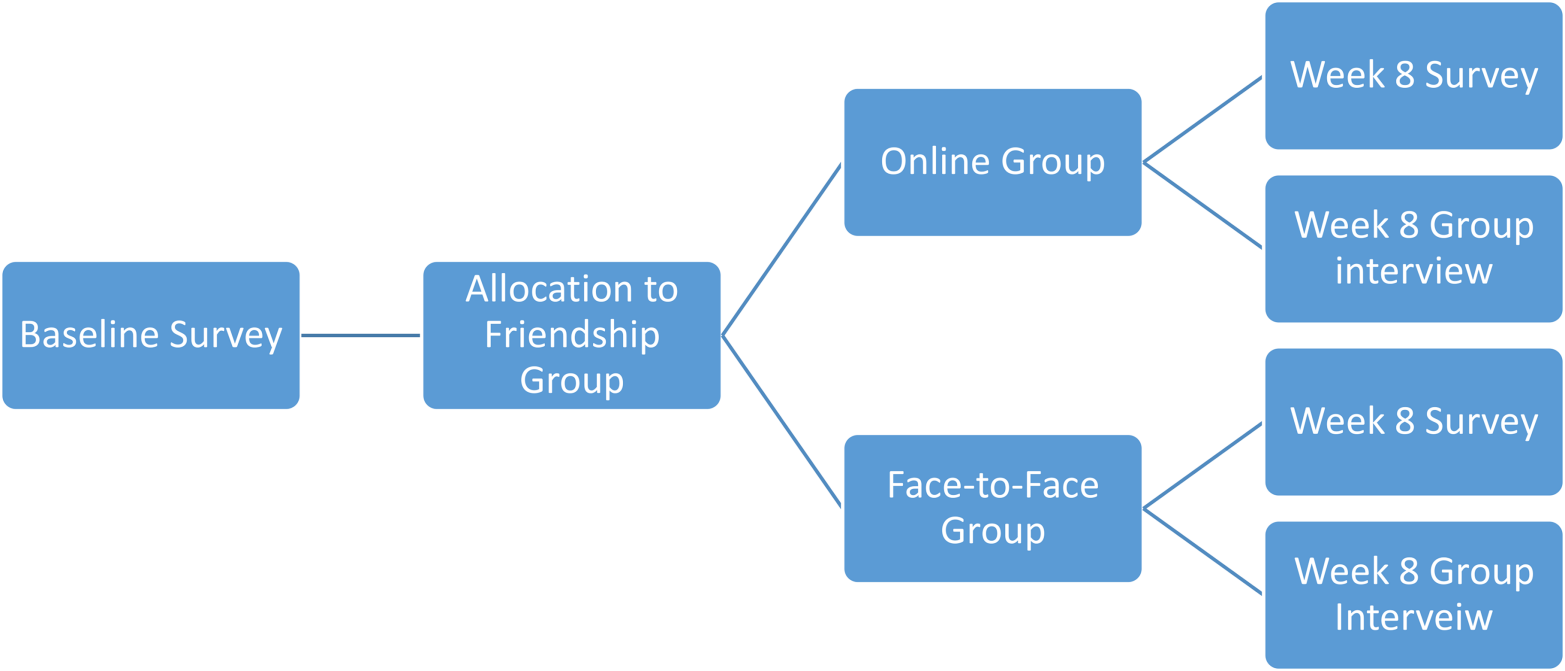
Allocation to online and face-to-face friendship groups.

Where possible, allocation to online or face-to-face groups was based on patient preference although it was explained to patients (before consenting) that this was dependent on availability. Once initial screening data was available, participants were contacted (via telephone) and asked to confirm their willingness to attend as well their preference for online or face–to-face groups. Given the resources available, a maximum of 60 participants could be recruited into the study. Group allocation was sequential and non–random (see Figure 2 below). Informed consent was obtained from everyone taking part in the study.

## Quantitative outcomes

The selection of outcome measures for this study was carefully considered to ensure relevance and appropriateness for the Cambodian context, as well as to comprehensively capture the impact of the intervention on mental health outcomes. Each measure was chosen based on its validity, reliability, and cultural relevance to the target population. As indicated above, all participants in the study completed a screening survey to determine suitability. This included some basic socio-demographic information as well a series of validated psychometric scales described below.

### Primary outcome measure

The primary outcome measure was Kessler-10 (Kessler et al., 2002) which is well suited to the Cambodian context and has been translated into Khmer and validated (38). Kessler-10 measures psychological distress and scoring ranges are as follows - likely to be well (score < 20), likely to have a mild distress (score = 20–24), likely to have moderate distress (score = 25–29) and likely to have a severe distress (score ≥ 30) (39).

### Secondary measures

#### Primary care PTSD screen for DSM-5

The PC-PTSD-5 (49) is a five-item clinician administered screen that identifies individuals with probable PTSD. It has been used widely in primary care settings and begins by asking the individual whether they have been involved in any potentially traumatic event. Validation studies have demonstrated that answering “yes” to three out of five questions is optimally sensitive to probable PTSD (50).

#### Pathological worry: the 3-item Penn State Worry Questionnaire (PSWQ-3)

The 3-item Penn State Worry Questionnaire (PSWQ-3) issued to measure pathological worry. The PSWQ-3 has comparable internal consistency and validity to the longer 16-item PWSQ, (42). The Cronbach's Alpha for the present study was .91; (51).

#### Facets of mindfulness: Chinese version of the cognitive and affective mindfulness scale — revised – (Ch-CAMS-R)

The acceptance and present focus subscales of this tool were combined to give an overall facets of mindfulness score, ranging from 4 to 16. The Ch-CAMS-R has been found to obtain good levels of reliability, validity and factor structure as the original CAMS-R (43) It was also found to have good convergent validity with the DASS-21 (43). The Cronbach's Alpha for the 4-item facets of mindfulness scale for the present study was .89.

#### Rumination reflection questionnaire

Rumination subscale (52) is a 12-item scale which measures the extent to which participants are disposed to engage in repetitive thinking about their past (rumination). Higher scores on the RRQ-rumination indicate higher levels of rumination. Scores on this measure range from 12 to 60. Trapnell and Campbell (52) reported a high coefficient alphas (Rumination = .90). For the current study it was.89.

## Statistical analysis

The data were screened for missing values and any error cases, such as extreme outliers. There were no missing values or error cases on any of the outcomes. The Wilcoxon Signed-Rank test was used to assess changes in pre-post scores for psychological distress, PTSD, worry, rumination and facets of mindfulness. With relatively small group sample sizes, differences between group allocations could confound intervention outcome measurement and it is therefore important in such circumstances to compare baseline differences between groups during the analysis (53). In order to do so, this study used analysis of covariance (ANCOVA), as this method increases the power of analyses of group allocations by reducing any unintentional baseline differences due to the allocation process, which increases a study's capacity to obtain a valid estimation of the intervention effect between groups (54, 55). In order to support balance in prognosis, intention-to-treat analysis was also employed (56). This allowed a pragmatic estimate of the benefit of the friendship group, rather than of its potential benefit in patients who receive treatment exactly as planned to be attained (56). No *p*-value adjustment was be made for multiple comparisons, as controlling for Type 1 error in this manner is likely to increase the chances of Type 2 error (57).

## Qualitative outcomes

Qualitative data was used by the research team to understand the feasibility and acceptability of FG's from the perspective of those who engaged in the intervention. Five focus group interviews were conducted 1–2 weeks post-intervention - four with FG participants (*n* = 21) and one with FG facilitators (*n* = 8). Audio recording took place during each interview, and this was transcribed verbatim.

## Data analysis

Given the small sample size and non-randomized design of this feasibility study, the statistical analysis is presented tentatively. Descriptive statistics were used to summarise baseline participant demographics and psychometric scores. Sum scores were determined for each construct within the screening survey. Wilcoxon Ranked Signed tests and two-way ANCOVAs were conducted to compare baseline and post-intervention scores for primary and secondary outcome measures.

Focus group data were analysed thematically (58) and followed a semi structured format that included core topics concerning – (1) general acceptability of the friendship group; (2) barriers to engagement (practical, material or psychological); (3) attitudinal or behavioural change during or post engagement; (4) facilitators or inhibitors of intervention effectiveness. Initially, transcripts from focus group interviews were read multiple times for familiarisation. Open coding was conducted on each transcript and labels that shared similarities were sorted into clusters and themes by two members of the research theme before being discussed among the wider research team. Through collaborative discussions, these codes were refined and grouped into overarching themes. The themes were then reviewed and defined to ensure they accurately represented participants' experiences. This systematic approach ensured a comprehensive analysis of the qualitative data.

## Results

*RO1: To compare uptake and retention between face-to-face and online delivery, aiming for at least 70% enrollment of eligible participants and a dropout rate not exceeding 30%*.

**Figure 3:**
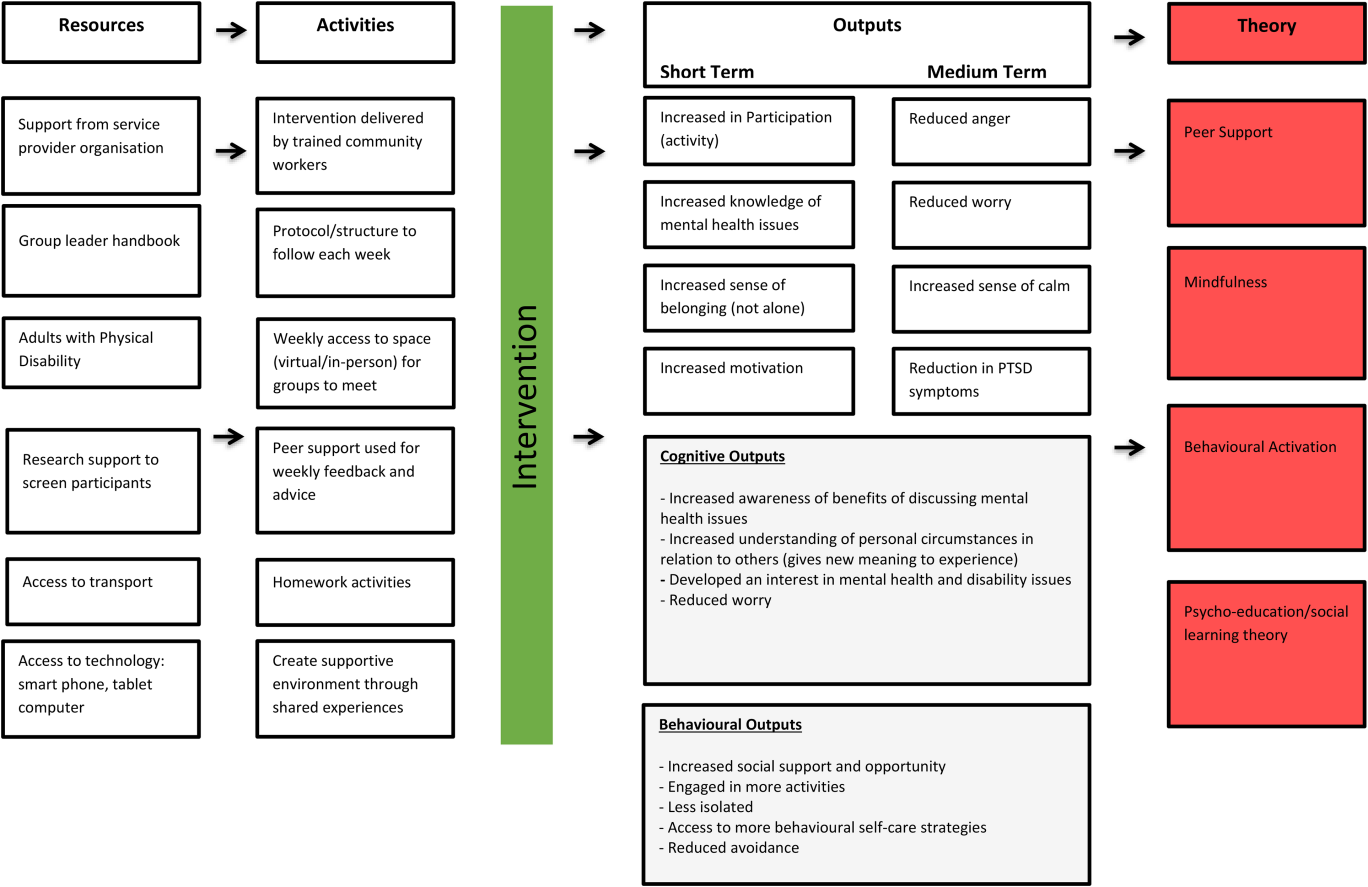
Logic model of development and implementation of friendship group intervention.

Following screening, 52 met inclusion and were invited to attend weekly FG's. Of those who had consented and agreed to take part, 22 were allocated across the two online groups and 19 participants were allocated across the two face-to-face groups (see Figure 3). Post-intervention, data was available for 18 participants in the online group (8 females and 10 males) and 14 participants in the face-to-face group (8 females and 6 males). This resulted in an uptake of 78% and an overall attrition rate of 22% with 26% dropout in face-to-face groups and 18% in online groups (Table 5).

**Table 5:** Friendship group outcomes (Pre/post-test).

	Friendship group total (*n* = 41)	Face to face group (*n* = 19)	Online group (*n* = 22)
Psychological distress – K-10 scores (pre)	21.7	24.1	19.6
Psychological distress – K-10 scores (post - ITT)	16.5	18.05	15.1
PTSD (pre)	25 participants with symptoms mean = 4	12 participants with symptoms mean = 4	13 participants with symptoms mean = 4
PTSD (post - ITT)	18 participants with symptoms mean = 3.28	7 participants with symptoms mean = 3.14	11 participants with symptoms mean = 3.25
Pathological worry (pre)	5.5	6.6	4.5
Pathological worry (post - ITT)	3.6	3.8	3.4
Rumination (pre)	36.7	37.1	36.4
Rumination (post - ITT)	35.9	35.9	35.9
Facets of mindfulness (pre)	9.27	10.5	8.2
Facets of mindfulness (post - ITT)	8.5	9.1	8

*RO2: To explore the potential benefit of weekly friendship groups in improving psychological distress*.

A Wilcoxon signed-ranks test showed a statistically significant decrease in psychological distress scores [*Z* = −3.808, *p* < .001] from pre [Mdn = 20, IQR = 16.5–25.5] to post [Mdn = 16, IQR = 14–18.5] Friendship group intervention. A Wilcoxon signed-ranks test also showed a statistically significant decrease in PTSD scores [*Z* = −2.239, *p* < .025] from pre [Mdn = 4, IQR = 3–5] to post [Mdn = 3, IQR = 2.75–4] Friendship group intervention. There was also a statistically significant decrease in worry scores [*Z* = −3.904, *p* < .001] from pre [Mdn = 5, IQR = 3.5–6.5] to post [Mdn = 3, IQR = 3–4] intervention. There were no statistically significant changes in facets of mindfulness [*Z* = −1.71, *p* < .09] or rumination [*Z* = −1.9, *p* < .06] from pre to post Friendship group.

*RO3: To compare pre to post outcome data from those who attended the face-to-face group and those who attended the online group*.

There were no significant group differences between the face to face and online groups in psychological distress [*F* (1, 39) = 2.38, *p*** = 0.13, *η^2^* = .06], PTSD [*F* (1, 13) = .04, *p*** = 0.84, *η^2^* = .003], worry [*F* (1, 39) = .15, *p*** = 0.7, *η^2^* = .004], rumination [*F* (1, 39) = .23, *p*** = 0.63, *η^2^* = .006] and facets of mindfulness [*F* (1, 39) = .66, *p*** = 0.42, *η^2^* = .017] from pre to post Friendship programme.

## Qualitative findings

*RO5: To train (task-shift) local Cambodia staff that work in the Cambodian School of Prosthetics and Orthotics to deliver a weekly “friendship group” intervention for stress, low mood, and anxiety*.

*RO6: To gather qualitative feedback from participants and facilitators to understand the acceptability and potential barriers to the intervention* .

**Figure 4:**
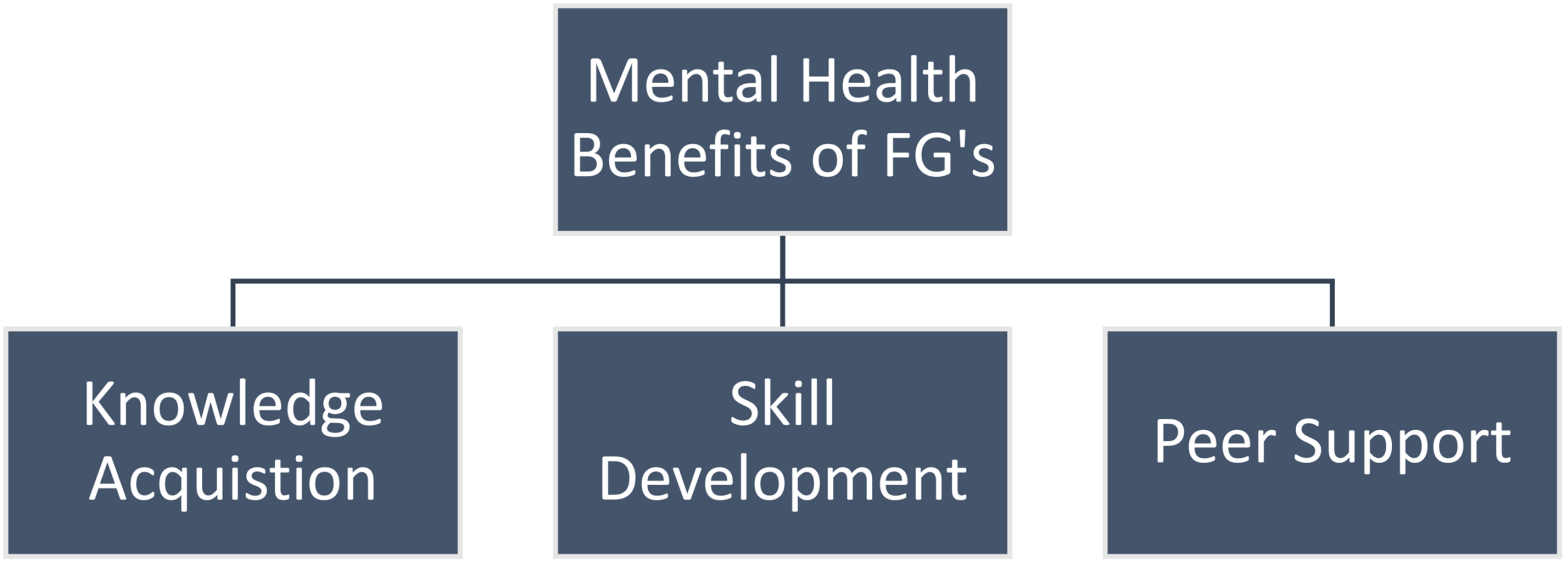
Themes and Sub-themes.

Five focus group interviews took place involving a total of 29 participants. Four focus groups took place with FG members only (*n* = 21) and one focus group was for facilitators only (*n* = 8). Several interconnected themes emerged during these interviews. These included the mental health benefits of Friendship Groups as conceptualised through knowledge acquisition (psycho-education and learning from others), skill development (mindfulness practices and learning to talk about feelings) and peer support. Issues of intervention acceptability and comparison between online and face-to-face support were also highlighted (Figure 4).

### General impact on mental health

Participants attending online or face-to-face friendship groups were largely positive regarding the overall mental health benefits. Of particular importance, was the benefit of mindfulness based techniques applied within the FG's with participants citing increased feeling of calmness over of the course of the intervention. As one participant suggested, “*the advantages to joining this group is we can do meditation to calm down our mind [and] not to get angry” (Group 3, Participant).* Others noted*, “in this course first we learn [meditative] exercises and apply them in our practice to calm ourselves” (Group 2, Participant).* These increased feelings of calmness were often conveyed in the context of previous issues with anger or sadness. For example,

*“We can control the mind and reduce anger in the past; I was very upset when I was amputee for one leg” (Group 1, Participant)*.

Another remarked that,

*“Before, it was different, after [I] go through meditation, it calms down, it is not one hundred percent, but 70 to 80 percent, so yeah. It reduces my anger much more than before” (Group 3 Participant)*

FG facilitators supported this, observing reduced anger and calmness over the course of the intervention “[if] they have difficulty with sleeping or if they have a problem with their failings and some of them have aggression…they could calm down often by just doing meditation with us” (Facilitator Focus Group).

### Knowledge acquisition and skill development

Group members discussed the new knowledge they had gained as an important benefit of the FGs in relation to their mental health. This included increased knowledge regarding mental health issues as well as learning new skills and techniques for self-care. They also appeared to frame FG's as a training course rather than a mental health support group. The sharing of knowledge within FG's appeared to take place organically and evolved throughout the 8-week intervention. As one participant noted, “*the benefit we get is knowledge for knowing society and the other difficulties each person with a disability has experienced” (Group 2, Participant).* A further benefit of this process was the focus on applying this new knowledge outside of the group. For example,

*“So I took this mental health course [and] I got better than before. I was able to educate myself mentally and when I go home, [I] take that experience to explain to my family.[that] this is a mental health issue. This course is all good and this learning is not just with talking, it is applicable for life, it is applied to make us speak well in words and gestures (Group 1, Participant)*.

Participants also spoke positively about learning new skills and techniques during FG meetings. This mainly included discussion regarding the mindfulness based techniques incorporated at the beginning and end of each session

*“…the meditation is the same from one day to the other day [and] it has led to calmness. When we think of something or something is not going well, we need to remind to this meditation and calmness” (Group 2, Participant)*.

Other participants supported this view during interviews,

*“For me before, I have felt a lot of anger and sadness, after I have this training it made me feel better and relief with less violence of pain in my heart and good to practice mediation too…since these two months, I feel better with business and the family is better too”. (Group 1, Participant)*.

### Peer support

The role of other group members emerged as a key theme during interviews. Chief among them was the sense that others have experienced similar difficulties,

*“to participate we know each other, we share, we listen to each other…there is only me as a person with disability in the village [and] I don't know who I can discuss with. When talking with others they discriminate…they don*’*t know our feeling as people with a disability” (Group 2, Participant)*.

Gaining the knowledge that there were others who had similar life experiences also appeared to encourage openness and discussion.

*“For me, there is gaining experience and knowledge from my participating…. [there are] many of us with a disability who are faced with so many struggled and challenges.. It*’*s not until now that I’m happy to have group discussion, that we get to know each other (Group 3, Participant)*.

Peer support appeared to provide the context in which to share information and describe experiences. As such, participants noted that they were able to develop their communication skills and ability to discuss their difficulties outside of the group “*it makes my life better and [I’m] learning to communicate with neighbours and within the family members is better” (Group 4, Participant).* In addition to this, participants noted using some of the practical advice provided by other members. One participant remarked that “*I have gained a lot of knowledge from all my uncles and aunts, and I have practiced a lot, and I have been able to overcome some of my fears”.* A further example of this was given when one participant spoke of how he was using the knowledge from his peers when dealing with anger – “*as the old person [elder] says…if angry get a stick… when you [find] the stick your anger will be gone” (Group 2, Participant)*. This view was also supported by FG facilitators who noted that –

*“…some come to understand that their difficulties are not really difficult because they compare to another participant that is more difficult….so they start to think and they feel like ‘okay, I have difficulties but not difficulties like you’…so they try to ‘get involved’ and try to enrich each other to get the feeling of a good vibe” (FG Facilitator Focus Group)*.

### Acceptability: online vs. face-to-face comparison

There appeared to be high levels of acceptability across participants in both online and face-to-face FG's with similar (positive) views recorded in relation to gaining new knowledge and the ability to connect with others. After the third week of FG's there were no further dropouts within either face-to-face or online groups. However, it was apparent that those in the online group experienced some additional barriers, such as intermittent connection issues. For example,

*“…it was difficult at times to have a conversation between facilitator and us because the internet was interrupted and lost connection (Group 4, Participant)*

*“…we meet every week [but] because the participants are far away from the internet…sometimes, we cannot hear each other” (Group 3, Participant)*.

Group facilitators also referenced this barrier with one noting there is “…difficulty accessing the technology so if you contact them regarding zoom or Microsoft Teams it is really hard for us to call…because if they are living in the province the network is not really stable”. The immediate impact of the connection issues was that online groups often had less time to take place. As one participant described, “…we usually know that online is not smooth at all…because sometimes we heard noise from everywhere and inside the meeting, so it wastes time” (Group 3, Participant). This led to feelings that there was limited time to explore issues in any depth, with one participant suggesting that “because our time is not favourable enough…we did not share all [of] it (Group 4, Participant). This view was supported by others who claimed that “for me, I think if there is a problem, it may be good to go deeper [into] this mental health issue…yes, go deeper and go deeper” (Group 4, Participant). In addition, one participant noted that the ability to hide your face online made it difficult at times to connect with others “I could see only one person at times as others hide their face” (Group 3, Participant). No such issues regarding depth and length of session where raised by participants in face-to-face FG's.

Some of the difficulties highlighted by participants were also raised by FG facilitators. This chiefly concerned online connection issues as well as ensuring that participants were dialling in from suitable environments with little background noise. One facilitator remarked that “*some of them were joining on time but some people had problem with joining… it means their starting process has become delayed so then we end up using lots more time than expected”.* In order to address these issues facilitators would send out reminders 30 min and then 15 min in advance of the meeting starting. They also suggested additional orientation sessions for new members in advance of meetings to test connection issues *e.g., “if we can set up a small session on how to use which is convenient and easy to use so that every participant can join into the group”.* However, they did stress that younger group members had fewer difficulties using the technology e.g.,

*“…in our online group some of them are quite young so they are quite good with the technologies [however] some of the participants are coming from a more rural area and they are quite elderly people…so that makes some difficulty in using all those things” (FG Facilitator Focus Group)*

Background noise was highlighted as an issue across both online and face-to-face groups and the importance of finding a quiet location was emphasised – “*Sometimes they slip off the camera, so you don't know what is going on. And then sometimes they have noise… they will ring during meditation sometime and sometime a song of birds, chicken, people.” (FG Facilitator Focus Group).* In spite of these additional barriers, online group members and facilitators welcomed the availability of such a service as there was little in the way of local service provision.

*“The communication through online is good because now is the period of Covid-19 but we can meet and consult through online. This is a positive point but like what my sister has just said, a negative point is that online communication is just a little affected as we rely on the internet. So while we can't meet face-to-face but we can discuss through online” (Group 3, Participant)*

In addition to this, FG facilitators spoke positively about their own experience of the intervention and how it has personally benefitted them. For example -

*“…the thing that made me feel good as [having] the time to participate in the group and listening to the participant directly and getting their experiences” (FG Facilitator Focus Group)*

*“…it has been a real benefit for me, and I think the other facilitator as well…being involved…I can like learn from the training with regarding to the friendship groups and how to facilitate and also regarding how to [deal] with mental issues” (FG Facilitator Focus Group)*

FG Facilitators were also positive regarding an expansion of the service in future, with one stating that

*“…in my own opinion I think if we got more of these kinds of friendship groups set up, I think it would be beneficial, I don*’*t think it would be a problem. I think it would be very beneficial for people, these facilities, that they would have a chance to say about their experience and also let out what is on their mind to the people with similar experiences that they might learn from each other” (FG Facilitator Focus Group)*

Others stated that they would like to see

*“…more friendship groups being conducted around Cambodia and bringing on more facilitators because now we have got some training, we feel more confident after eight weeks of practice” (FG Facilitator Focus Group)*

*RO7: To develop a logic model to inform intervention development and the evaluation of the intervention at a larger scale trial.*

The logic model for our Friendship Group Intervention was developed following data analysis and using the Theory of Change (ToC) framework (59) to map out the intervention's resources, activities, and expected outcomes. Through providing an in-depth understanding of how this intervention is theorised to work and which components are the most important in achieving impact, we aimed to reach a stronger external validity (60, 61). The finalised logic model illustrates the pathways from resources and activities to short-term and medium-term outcomes, grounded in theories of peer support, mindfulness, behavioural activation, and psycho-education/social learning theory.

## Discussion

**Figure 5:**
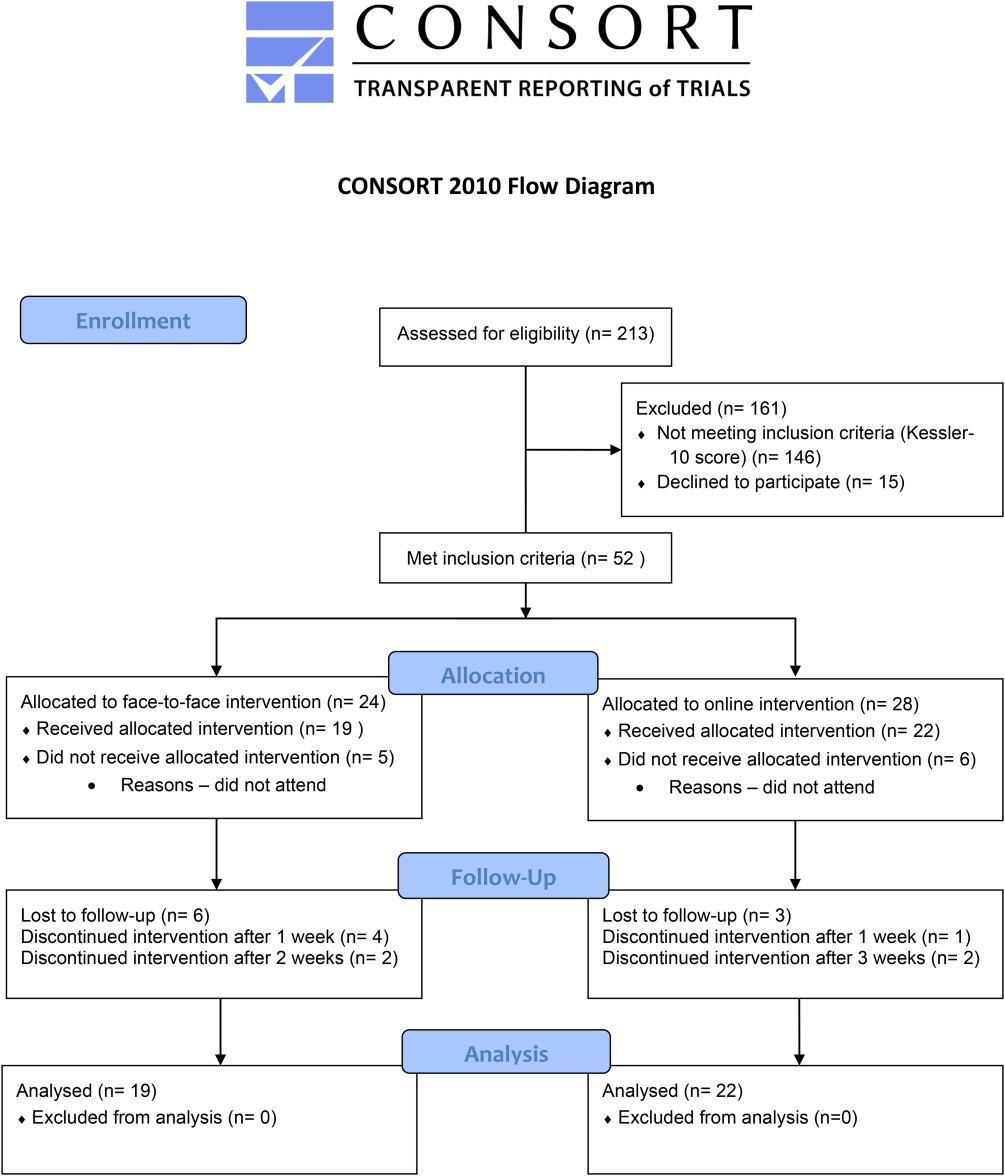
CONSORT flow diagram.

This intervention development study has shown promise in relation to a number of key areas. Initial findings show reduced psychological distress, levels of worry and PC-PTSD-5 scores for those attending the 8-week FG intervention (see logic model – Figure 5). There were no significant differences in primary outcome scores across online and face-to-face groups. Rumination and levels of facets of mindfulness showed a mean reduction however, these were not statistically significant. Overall, the intervention achieved an uptake rate of 78%, with an attrition rate of 22% across all four groups. Interestingly, the dropout rate was higher in the face-to-face groups (26%) compared to the online groups (18%). The lower attrition rate in the online Friendship Groups (FGs) was surprising given the technological barriers; however, this may reflect the scarcity of local in-person mental health services. As a result, the study's uptake rate of 78% exceeds the acceptable threshold of 70% for feasibility studies (36, 62). Additionally, the retention rate of 78%, with a dropout rate of 22%, falls within the acceptable range of up to 30% (63, 64). These metrics indicate that our intervention is both feasible and acceptable to participants.

Quantitative data indicated positive outcomes in relation to worry and PTSD symptoms. This was explored within focus group data which indicated a potential combination of factors, such as knowledge acquisition, skills development, and peer support. Our findings align with those of Ibrahim et al. (29), who identified that organizational culture, adequate training, and clear role definitions are critical for the successful implementation of peer support work. Gaining new knowledge regarding the impact of mental health issues as well as learning skills to support self-care (e.g., mindfulness practice and communicating how they feel to others) appeared to help to build confidence among group members. The peer support component provided a useful context in which these experiences and new knowledge could be shared and validated. As such, the focus on shared learning and practical advice created an environment more akin to a training course than a mental health intervention (“*after I have this training”* and *“so I took this mental health course”*). This may illustrate how change is influenced through social learning theory (SLT) (65) considering that support networks, the environment and context within which support is provided have proven to be vitally important in the success of this intervention. In turn, this may have increased the acceptability of the FG intervention by reducing perceived stigma.

Common emotions cited by group members following the intervention were increased feelings of calm and reduced feelings of anger. This supports some of the quantitative data that showed reductions in stress as well as PTSD, anger being a common emotion (66, 67). Cooper et al. (31) highlighted the effectiveness of peer support in improving depression symptoms, self-efficacy, and recovery, which parallels our observation of reduced psychological distress and worry among participants. In addition, while the intervention did not target PTSD symptoms specifically, the shared experience of a physical disability may have enabled some participants to re-evaluate their own experience and the impact it has had. This new information may have reassured them that are “not alone” and bring new meaning to the event – a key step during PTSD treatment (68).

Quantitative data showed no significant differences in psychological distress scores across both delivery formats, with both showing positive effects. Qualitative data did reveal some additional barriers in regard to online delivery with disruptions in internet connection and background noise meaning that groups sometimes felt rushed and less opportunity for in-depth discussion. In most cases however, solutions to connection issues where solved over time as members got more familiar with the technology and facilitators took a more active role in encouraging attendance in advance of meeting start time. Interestingly, these initial issues did not seem to affect retention and attendance, with attrition in online groups (14%) lower than face-to-face groups (32%). An additional positive of online groups was the ability to access services that were unable available locally and this was acknowledged by several members.

There appeared a high level of acceptability across group members and facilitators during the study indicating potential feasibility of a larger scale research project. Those who attended online and face-to-face FG's reported positive outcomes and some indicated a willingness to expand the service or continue on after the intervention period. Lyons et al. (30) found that group peer support interventions lead to significant improvements in personal recovery outcomes, such as hope and empowerment, which supports our findings of increased participant confidence and reduced psychological distress**.** The label of “Friendship Group” rather than “mental health support group” appeared culturally appropriate and the incorporation of techniques, such as mindfulness, behavioural activation and peer support appear to translate well within the local context. FG facilitators grew in confidence as the intervention progressed and appeared to find it a beneficial experience. There were no reports of unnecessary or additional burden or harm and this included both the intervention itself as well as the survey questionnaire participants were asked to complete.

### Implications for future research

The findings from this study suggest important implications for future research in peer-led mental health interventions, especially in low-middle income countries like Cambodia. The observed reductions in psychological distress, worry, and PTSD symptoms indicate the potential value of peer support models in this context. Future research should use more robust experimental designs, such as RCTs, and larger sample sizes to strengthen the evidence base and ensure generalisability. Addressing technological barriers and ensuring participants' familiarity with online platforms is crucial. Research should explore effective ways to deliver online interventions, particularly in areas with limited internet connectivity, and provide quiet spaces for both face-to-face and online sessions to minimize disruptions. The framing of Friendship Groups as training courses may reduce stigma and increase participation, an area worth further exploration. Expanding the content to include more skills development and minimizing gaps between initial screening and intervention delivery are also important. The interconnected nature of new knowledge and peer support in reducing societal stigma was significant, suggesting that these components should be emphasized to enhance the intervention's effectiveness. Lastly, training community-based workers to deliver the intervention is cost-effective and scalable, making it viable for other low-resource settings.

### Strengths and limitations

The strengths of this study include the development and implementation of low cost, community based mental health support groups within the Cambodia context. The intervention appeared to be culturally appropriate and acceptable and was delivered by individuals with no prior mental health training. The intervention was also embedded within a pre-existing physical orthopaedic service and which further reduced costs as well as targeted a population of increase risk of mental health issues. Limitations in include the relatively small sample, non-controlled design and lack of random allocation to groups. Qualitative data was also translated and transcribed from Khmer into English by NGO partners and thus aspects of the interview data may have been lost of misunderstood. As such, the results should be interpreted with caution.

## Conclusion

In conclusion, the friendship group intervention delivered in both online and face-to-face formats appears feasible and acceptable for adults with physical disabilities within the Cambodian context. Initial data revealed positive findings in terms of reduction in psychological distress, worry and PTSD symptoms as well increased feeling as calm. The combination of new knowledge and skills within the context of peer support appeared as the main drivers behind the benefits that members experienced. While the role of wider societal stigma wasn't explicitly acknowledged by group members, in relation to both physical and mental health disability. The interconnected nature of new knowledge and peer support appear to reduce its impact. This may have implications for future recruitment and retention. By adopting a peer support model and training community-based workers, the research team was able to deliver this mental health intervention at a much lower cost. Future studies would do well to build upon this work by using a more robust (controlled) experimental research design and recruiting a larger sample. Moreover, careful consideration of online platforms for connectivity and familiarity among participants is necessary, alongside ensuring a quiet space for both face-to-face and online group delivery. Expanding the length of the initial orientation session for online members and framing Friendship Groups as training courses may lessen associated stigma and improve engagement. Including additional content around skills development and minimising gaps between initial screening and intervention delivery are also important considerations.

## Data Availability

The datasets presented in this article are not readily available because the datasets generated and/or analysed during the current study are not publicly available due to the requirement from the ethics review committee but are available from AM on reasonable request. Requests to access the datasets should be directed to alanmaddock@rcsi.ie.
